# Neural correlates of blood flow measured by ultrasound

**DOI:** 10.1016/j.neuron.2022.02.012

**Published:** 2022-05-18

**Authors:** Anwar O. Nunez-Elizalde, Michael Krumin, Charu Bai Reddy, Gabriel Montaldo, Alan Urban, Kenneth D. Harris, Matteo Carandini

**Affiliations:** 1 UCL Institute of Ophthalmology, University College London, London WC1E 6AE, UK; 2 Neuro-Electronics Research Flanders, 3001 Leuven, Belgium; 3 Vlaams Instituut voor Biotechnologie (VIB), 3000 Leuven, Belgium; 4 imec, 3001 Leuven, Belgium; 5 Department of Neuroscience, KU Leuven, 3000 Leuven, Belgium; 6 UCL Queen Square Institute of Neurology, University College London, London WC1E 6AE, UK

**Keywords:** electrophysiology, ultrasound measurements, mice, hemodynamic coupling

## Abstract

Functional ultrasound imaging (fUSI) is an appealing method for measuring blood flow and thus infer brain activity, but it relies on the physiology of neurovascular coupling and requires extensive signal processing. To establish to what degree fUSI trial-by-trial signals reflect neural activity, we performed simultaneous fUSI and neural recordings with Neuropixels probes in awake mice. fUSI signals strongly correlated with the slow (<0.3 Hz) fluctuations in the local firing rate and were closely predicted by the smoothed firing rate of local neurons, particularly putative inhibitory neurons. The optimal smoothing filter had a width of ∼3 s, matched the hemodynamic response function of awake mice, was invariant across mice and stimulus conditions, and was similar in the cortex and hippocampus. fUSI signals also matched neural firing spatially: firing rates were as highly correlated across hemispheres as fUSI signals. Thus, blood flow measured by ultrasound bears a simple and accurate relationship to neuronal firing.

## Highlights

• Neural activity was recorded during ultrasound imaging (fUSI) of blood flow in mice
• fUSI signals matched the smoothed firing rate of local neurons, especially inhibitory
• The ∼3 s smoothing filter is the stereotypical hemodynamic response of awake mice
• Spatiotemporal correlations in firing rates largely explain correlations in blood flow

## Graphical abstract

**Figure:**
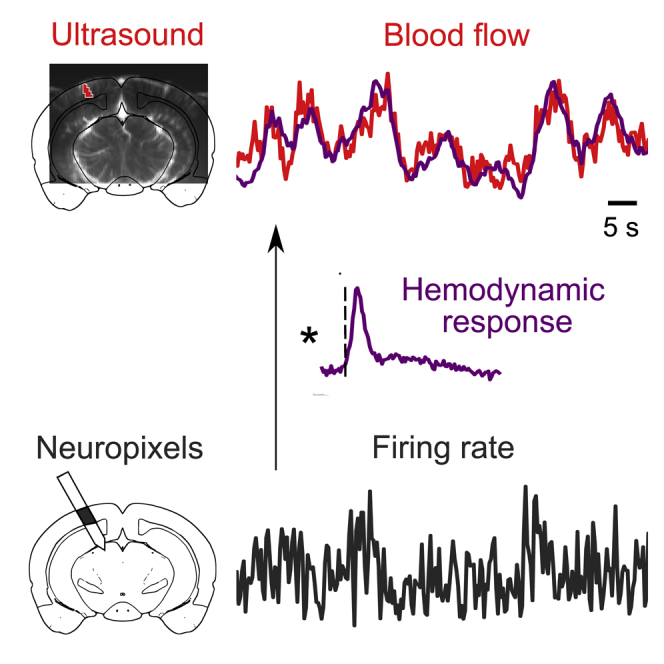

## Teaser

Nunez-Elizalde et al. simultaneously measured neural activity with electrodes and blood flow with functional ultrasound imaging (fUSI) in awake mice. Their results establish that blood flow bears a simple and accurate relationship to local neuronal firing at frequencies <0.3 Hz and validate fUSI as a tool to infer neural activity.

## Introduction

Functional ultrasound imaging (fUSI) is an appealing method for studying brain function because it estimates changes in the cerebral blood volume with high resolution, resolving spatial features ∼100 μm in size to a depth of ∼2 cm (Deffieux et al., 2018; Edelman and Macé, 2021; Macé et al., 2011; Rabut et al., 2020). It is thus used to study how the activity of brain regions depends on sensory stimuli, internal state, and behavior in species ranging from mice (Aydin et al., 2020; Boido et al., 2019; Brunner et al., 2020; Ferrier et al., 2020; Koekkoek et al., 2018; Macé et al., 2018; Sans-Dublanc et al., 2021) to rats (Bergel et al., 2018, 2020; Gesnik et al., 2017; Macé et al., 2011; Osmanski et al., 2014; Provansal et al., 2021; Rahal et al., 2020; Sieu et al., 2015; Urban et al., 2015), to marmosets (Zhang et al., 2021), ferrets (Bimbard et al., 2018), and macaques (Blaize et al., 2020; Dizeux et al., 2019). In a small animal such as the mouse, fUSI can image the whole brain, yielding measurements that may parallel those obtained in humans with functional magnetic resonance imaging (fMRI).

However, the relationship between fUSI signals and neural activity is indirect: it relies on the physiology of neurovascular coupling, the physics of ultrasound sensing, and the mathematics of the subsequent signal processing. Neurovascular coupling links neuronal firing to changes in blood flow and volume (Attwell and Iadecola, 2002; Drew, 2019; Hamel, 2006; Hillman, 2014; Iadecola and Nedergaard, 2007; Nair, 2005; Pisauro et al., 2013; Turner et al., 2020; Winder et al., 2017). Movement of blood, in turn, causes a frequency shift in ultrasound echoes measured through power Doppler ultrasound sensing (Rubin et al., 1994, 1995). These power Doppler signals must then be distinguished from multiple, large sources of noise—such as tissue movement—through multiple steps of signal processing. These typically include temporal binning, power estimation, temporal high-pass filtering, and spatiotemporal clutter filtering by removing the largest principal components (Baranger et al., 2018; Demené et al., 2015; Macé et al., 2011, 2013). Small changes in this procedure can profoundly affect the inferred neural signals (e.g., Demené et al., 2015). However, this procedure has not been verified with simultaneous recordings of neuronal firing in the awake brain. It is unclear to what degree and at what temporal and spatial scales, fUSI signals can measure simultaneous neural firing.

At first sight, fUSI signals appear noisy, with large fluctuations over short time scales (e.g., >10% over a few seconds) that vary across trials (e.g., Brunner et al., 2020), and it is not clear to what extent these fluctuations are due to measurement error or to the underlying neural activity. Neural activity exhibits endogenous, ongoing fluctuations that are strongly correlated across neurons (Schölvinck et al., 2015) and hemispheres (Drew et al., 2019; Fox et al., 2006, 2007; Mohajerani et al., 2010; Shimaoka et al., 2019). Perhaps the apparently noisy fUSI signals reflect these structured fluctuations in neural activity. Indeed, fUSI signals resemble simultaneously recorded local field potentials (LFPs) (Aydin et al., 2020; Bergel et al., 2018, 2020; Sieu et al., 2015), which in turn reflect local neuronal firing (Buzsáki et al., 2012; Katzner et al., 2009).

Moreover, it is not clear whether fUSI signals reflect neuronal firing through a simple, constant relationship. Neurovascular coupling is approximately linear: hemodynamic signals can be predicted by smoothing firing rates with a hemodynamic response function (HRF) (Boynton et al., 1996; Cardoso et al., 2019; Devor et al., 2005; Drew, 2019; Heeger and Ress, 2002; Lima et al., 2014; Logothetis et al., 2001; Martindale et al., 2003; Pisauro et al., 2013). The next step might also be linear: fUSI signals can be predicted from hemodynamic signals (red blood cell velocity) through another transfer function, at least after trial averaging (Aydin et al., 2020; Boido et al., 2019). Because a series of linear operations is itself linear, the relationship between fUSI signals and neuronal firing may be linear. Furthermore, this relationship may vary across brains, brain regions, or brain states.

Here, we answer these questions with simultaneous measurements of spikes and fUSI signals. We performed these measurements in the awake brain because anesthesia impairs the function of inhibitory circuits (Haider et al., 2013) and degrades the mechanisms of neurovascular coupling (Pisauro et al., 2013).

## Results

**Figure 1:**
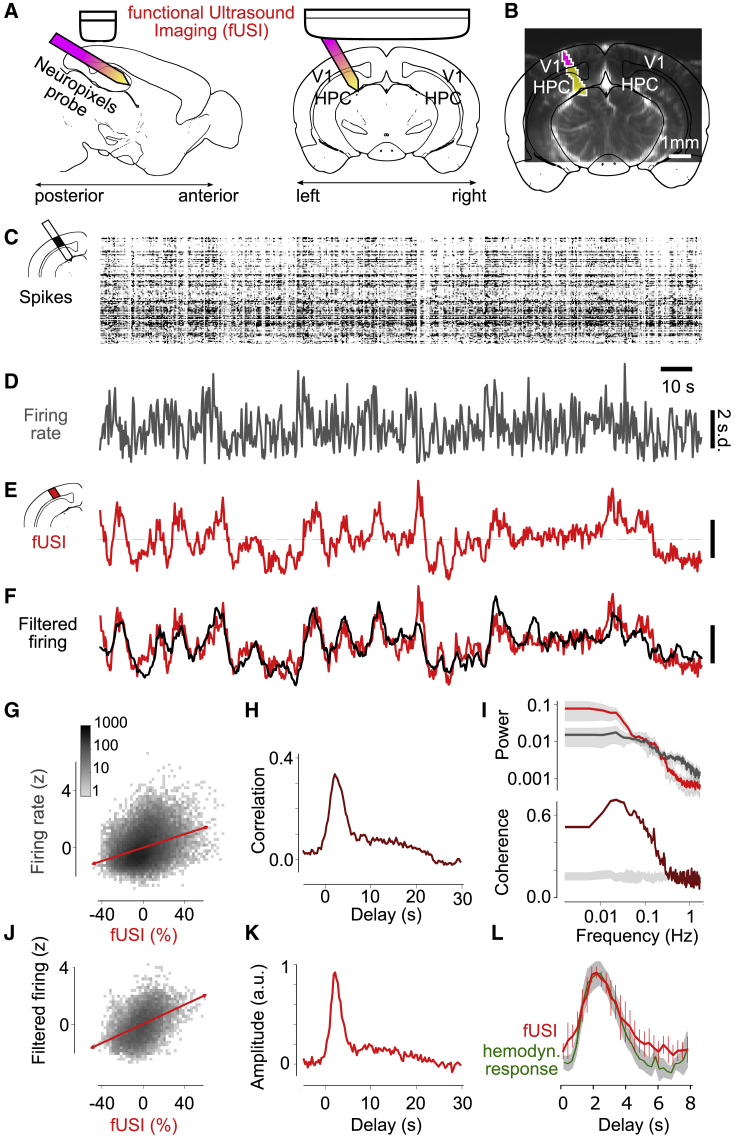
fUSI signal reflects temporally filtered firing rates during spontaneous activity (A) Schematic of simultaneous fUSI and electrophysiological recordings showing the primary visual cortex (V1) and hippocampus (HPC). (B) Coronal fUSI slice with the location of the Neuropixels probe passing through this plane (purple) and in front of it (yellow). (C) Spikes recorded in V1 in an example recording as a function of time and recording depth. (D) The resulting mean firing rates. (E) fUSI signal measured simultaneously in the same location (average over 51 voxels). (F) Smoothing the firing rates with the optimal filter (shown in K) yields good predictions (black) of the fUSI signals (red). (G) Comparison of fUSI signals and firing rates measured 2.1 s earlier (the optimal value), with best-fitting lines indicating correlation (red). 34 recordings in 5 mice. (H) Cross-correlations between firing rates and fUSI signals, averaged across 34 recordings in 5 mice. (I) Power spectra (top) and spectral coherence (bottom) of firing rates and fUSI, averaged across recordings. The gray bands in the top plot show 1 median absolute deviation (MAD). The gray band in the bottom plot shows coherence of randomly circularly shifted traces. (J) Comparison of fUSI signals and filtered firing rates. (K) Optimal linear filter across recordings obtained with cross-validation. Median of 34 recordings in 5 mice. (L) The filter (red) resembles the hemodynamic response function measured in awake mice (green, from Pisauro et al., 2013). Error bars show ± MAD of 34 recordings in 5 mice.

To record neuronal firing during fUSI, we inserted a Neuropixels probe (Jun et al., 2017) in a parasagittal trajectory while acquiring a fUSI image coronally (Figures 1A and 1B). Mice were awake and generally alert, as confirmed by pupil dilation and whisker movements (McGinley et al., 2015; Reimer et al., 2014) (Figure S1). They viewed a gray screen (to measure spontaneous activity) or flashing checkerboards (to measure visual responses). Recordings were repeated after moving the fUSI transducer ∼0.4 mm to an adjacent coronal slice (3–5 slices per session). At the end of a session, we located the probe in the fUSI images by extracting it while detecting its movement with fUSI (Figure 1B). We then processed fUSI signals with established procedures (Demené et al., 2015; Macé et al., 2011).

fUSI signals from the visual cortex during spontaneous activity resembled a delayed and smoothed version of the firing rate measured in the same location. After spike sorting, we computed the mean firing rates in all neurons (both single- and multi-unit clusters) recorded at the sites that intersected the fUSI slice (Figures 1C and 1D). This firing rate resembled the fUSI signal measured in the corresponding voxels (Figure 1E). The correlation between firing rates (delayed by 2.1 s) and fUSI signals was ρ = 0.34 ± 0.08 (median ± median absolute deviation [MAD], 34 recordings in 5 mice, Figure 1G). The cross-correlation peaked at a delay of 2.1 ± 0.3 s, with full width at a half-height of 3.6 ± 0.6 s (± MAD, 34 recordings in 5 mice, Figure 1H).

Firing rates and fUSI signals were best correlated at low frequencies. Their spectral coherence, i.e., their correlation as a function of frequency, was highest between 0.01 and 0.1 Hz, with a median correlation of 0.59 ± 0.03 (MAD, 34 recordings in 5 mice), and gradually fell to chance levels (coherence of 0.14 ± 0.03) at a frequency of 0.32 Hz (Figure 1I). These results indicate that low-frequency fluctuations in fUSI are related to neural activity, whereas fluctuations at higher frequencies might best be discarded.

The relationship between fUSI signals and firing rates was well described by convolution with a linear filter. We estimated the optimal filter that relates the two through convolution (Boynton et al., 1996; Pisauro et al., 2013), using cross-validated ridge regression (Hoerl and Kennard, 1970). Smoothing the firing rate with this filter yielded a prediction that closely matched the fUSI signal (Figure 1F). The filtered firing rates and the fUSI signals were highly correlated: in held-out data, the median correlation between the two was ρ = 0.49 ± 0.13 (MAD, 34 recordings in 5 mice, Figure 1J).

The filter relating fUSI signals to firing rates resembled the hemodynamic response function (HRF) characteristic of awake mice. As expected, the estimated filter peaked with the same delay as the cross-correlations (2.1 ± 0.3 s, median ± MAD), but it had a faster time course (cross-correlations are blurred by autocorrelations of the signals). Its full width at half-height was 2.9 ± 0.6 s (MAD, 34 = experiments in 5 mice, Figure 1K). This time course resembled the HRF measured optically in the cortex of awake mice (Pisauro et al., 2013; Figure 1L), though possibly with a longer tail (Aydin et al., 2020). The estimated filter, therefore, likely corresponds to the HRF of the awake mouse.

### Hemodynamic coupling across stimulus conditions and neural sources

**Figure 2:**
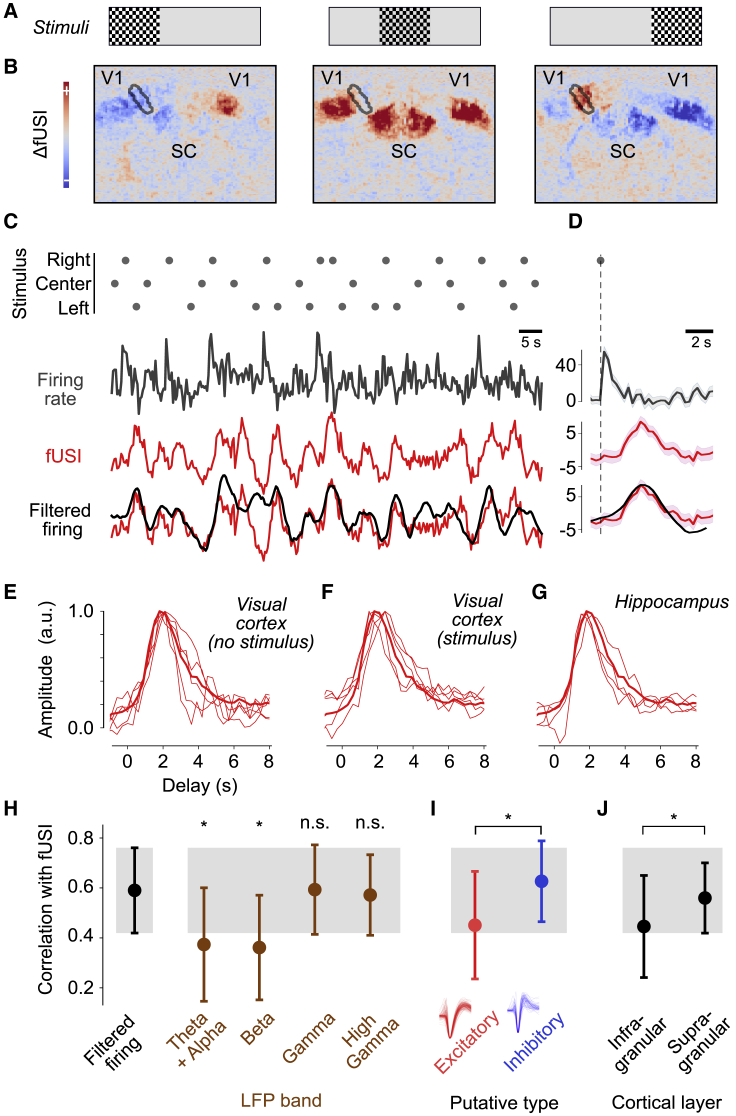
Constant hemodynamic coupling across stimulus conditions and neural sources (A) Flashing checkerboards were presented on the left, center, or right. (B) fUSI voxel responses to checkerboards, showing deviations from the mean activity. Black outline indicates the voxels traversed by the Neuropixels probe in V1. (C) Response to an example sequence of 30 stimuli (dots), showing firing rates in left V1 (gray), the corresponding fUSI signal (red), and the filtered firing rate (black). (D) Same format as (C), showing the average response to the right (optimal) stimulus. (E and F) The estimated HRFs for the visual cortex under spontaneous activity and visual stimulation for individual mice (n = 5) resembled the mean HRF computed across mice, areas, and conditions (thick curve). (G) Individual HRFs for hippocampus estimated across spontaneous activity and visual stimulation (n = 4) resembled the mean HRF (thick curve, same as in E and F). (H) Correlation between fUSI signals and LFP bands (n = 187 recordings across hippocampus and visual cortex, in 5 animals). Asterisks indicate significant differences between firing rates and LFP bands (p < 10^−12^). (I) Correlation between fUSI signals and putative excitatory and inhibitory neurons (n = 187 recordings). (J) Correlation between fUSI signals and infragranular and supragranular units recorded from the visual cortex (n = 100 recordings in 5 animals).

This simple linear relationship explained cortical fUSI signals also during visually driven activity. To evoke visual responses, we presented a sequence of flashing stimuli on the left, center, and right (Figure 2A). In this sequence, there was only a 2.5% chance that a stimulus would reappear consecutively in the same position. The typical interval between stimuli in the same position was >7 s and often longer, allowing fUSI signals to return to baseline between stimuli. An event-related analysis showed the expected representation of visual space in the primary visual cortex and superior colliculus (Brunner et al., 2020; Gesnik et al., 2017; Macé et al., 2018; Figure 2B). Just as with spontaneous activity, the fUSI signal was well predicted by smoothing the firing rate with the estimated HRF (Figures 2C and 2D). In held-out data, its median correlation with filtered firing rates was ρ = 0.55 ± 0.22 (MAD across 34 experiments in 5 mice).

The estimated HRF was similar across mice, stimulus conditions (driven versus spontaneous), and brain regions (visual cortex versus hippocampus). The HRFs measured in the visual cortex were similar across mice, both during spontaneous activity and during visual stimulation (Figures 2E and 2F). Moreover, they resembled the HRFs measured in the hippocampus (Figure 2G). To assess the similarity of the HRF, we compared the fUSI signals predicted while allowing different HRFs versus imposing a single average HRF (thick curve in Figures 2E–2G). We used cross-validation to avoid overfitting. The average HRF explained a similar fraction of the variance as the individual HRFs. Therefore, although the visual cortex and hippocampus show marked differences in vascular networks (Shaw et al., 2021), they have similar hemodynamic responses.

fUSI signals correlated equally well with neuronal firing rates and with LFPs in the gamma range. The LFP reflects the average neural activity in a local region (Buzsáki et al., 2012; Katzner et al., 2009). We measured its power in four frequency bands: 4–12 Hz (alpha and theta), 12–30 Hz (beta), 30–90 Hz (gamma), and 110–170 Hz (high gamma). As expected (Aydin et al., 2020; Lima et al., 2014), fUSI signals correlated best with LFPs in the gamma and high-gamma ranges (Figure 2H), with correlations not significantly different from those observed with firing rates (p = 0.57 and p = 0.08, paired t test). The two lower LFP frequency bands, instead, yielded significantly lower correlations (p < 10^−12^).

fUSI signals were best correlated with the activity of putative inhibitory neurons. We distinguished putative excitatory and inhibitory neurons based on their spike shapes (Barthó et al., 2004). fUSI signals correlated significantly better with the filtered firing of putative inhibitory neurons (ρ = 0.63 versus 0.45, p < 10^−12^, paired t test). This difference was not due to a larger number of spikes because the putative inhibitory neurons collectively fired fewer spikes. Indeed, the difference was still significant when we equated spike numbers by subsampling (p < 10^−12^).

In the cortex, finally, fUSI signals were best correlated with activity measured in supragranular layers compared with infragranular layers (ρ = 0.56 versus 0.44, p = 0.005, paired t test). Again, this effect was not due to larger numbers of spikes because supragranular neurons have lower firing rates (Sakata and Harris, 2009) and because the difference was still significant when we equated spike numbers through subsampling (p = 0.002).

### fUSI signals and firing rates are correlated across hemispheres

**Figure 3:**
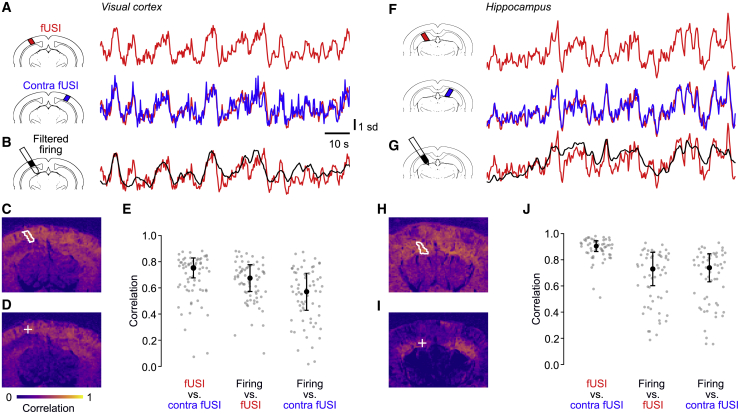
fUSI signals and firing rates are correlated across hemispheres (A) fUSI traces measured during spontaneous activity in an example recording, in an ROI in the left visual cortex (top) and in a symmetrical ROI in the right visual cortex (bottom). (B) Filtered firing rates measured simultaneously in the left ROI. (C) Correlations between the fUSI voxels in the left ROI (white contour) and all the other fUSI voxels. (D) Correlations between the filtered firing rates measured in the left ROI (plus sign) and all the individual fUSI voxels. (E) Correlations between fUSI signals in the left and right visual cortices (left) and between filtered firing rates and simultaneous fUSI signals in the same location in the visual cortex (center) and in the opposite hemisphere (right). Black dot and error bars show median ± MAD across n = 68 recordings during spontaneous activity and visual stimulation. (F–J) Same analyses for recordings where firing rates and fUSI were measured in hippocampus (n = 58 recordings).

Similar to BOLD fMRI signals (Desjardins et al., 2001; Fox et al., 2006, 2007; Macey et al., 2004; Murphy et al., 2009), fUSI signals have broad spatial correlations within and across hemispheres, allowing the use of fUSI to study “functional connectivity” (Ferrier et al., 2020; Osmanski et al., 2014; Rabut et al., 2019, 2020; Urban et al., 2015). Indeed, the fUSI signals measured in the left visual cortex during spontaneous activity correlated highly with signals in many other cortical and subcortical locations, even across hemispheres (Figures 3A and 3C). Correlations across hemispheres were as high as ρ = 0.75 ± 0.08 (median ± MAD across 68 recordings; Figure 3E, left). Similar results were seen in the hippocampus (Figures 3F and 3H), with bilateral correlations of ρ = 0.90 ± 0.04 (across 58 recordings; Figure 3J, left).

Accordingly, the filtered firing rates correlated not only with fUSI signals at the same location but also at other locations, including those in the opposite hemisphere. This can be seen for firing rates measured in the left visual cortex (Figures 3B and 3D): their correlations with contralateral fUSI signals were ρ = 0.57 ± 0.14, barely lower than correlations with ipsilateral fUSI signals (ρ = 0.68 ± 0.10, Figure 3E, center and right). Similar results can be seen for firing rates measured in the left hippocampus (Figures 3G and 3I), with correlations above 0.7 with fUSI signals measured in the hippocampus of either hemisphere (Figure 3J).

The strong spatial correlations seen in fUSI signals may thus be explained by fluctuations in neural activity. Indeed, ongoing neural activity has broad spatial correlations and is strongly bilateral, both during rest and during behavior (Mohajerani et al., 2010; Musall et al., 2019; Shimaoka et al., 2019). However, there is another possible source of spatial correlations: there may be hemodynamic fluctuations that are broad and bilateral but unrelated to neuronal activity (Drew et al., 2020; Turner et al., 2020).

### Bilateral firing largely explains bilateral fUSI signals

**Figure 4:**
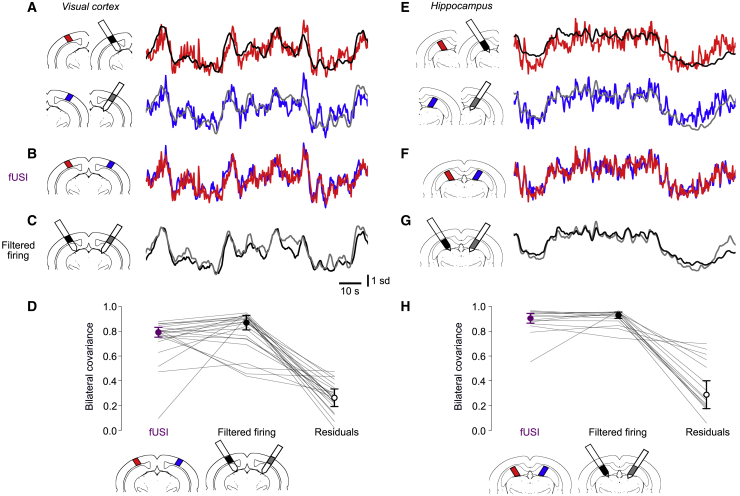
Bilateral firing largely explains bilateral fUSI signals (A) Example recordings from two Neuropixels probes inserted bilaterally, yielding simultaneous measurements of firing rates (filtered with the HRF, black and gray curves) and fUSI signals (red and blue curves) during spontaneous activity in left and right visual cortices. (B and C) Superposition of the bilateral fUSI signals (B) and of the bilateral filtered firing rates (C). (D) Covariance between left and right fUSI signals (left), filtered firing rates (middle), and residuals obtained by subtracting the filtered firing rates from the fUSI signals (right). Because fUSI signals and filtered firing rates are *Z* scored, their covariance equals their correlation. Dots and error bars indicate median ± MAD across 22 recordings (lines) in 3 mice during spontaneous activity and visual stimulations. (E–H) Same analysis for hippocampus (14 recordings in 3 mice).

To investigate the high bilateral correlations observed in fUSI, we performed simultaneous recordings with two Neuropixels probes. In three of the mice, we inserted two probes symmetrically, targeting bilateral locations in the visual cortex (Figure 4A). We could thus compare not only fUSI signals with filtered firing rates measured locally (Figure 4A) and with contralateral fUSI signals (Figure 4B) but also firing rates across hemispheres (Figure 4C).

Filtered firing rates measured in one hemisphere were highly correlated with those in the symmetrical position in the other hemisphere. This can be seen for filtered firing rates in the visual cortex (Figure 4C), which had a high bilateral correlation, ρ = 0.87 ± 0.06 (median ± MAD, across 22 recordings; Figure 4D, middle). These correlations in firing rates were not lower than those measured in fUSI signals ((paired t test p = 0.28, n = 22, Figure 4D, left). Similar results were seen in the hippocampus (Figures 4E–4G): the filtered firing rates measured in the left and right hippocampus were highly correlated, ρ = 0.93 ± 0.03 (median ± MAD across 14 recordings; Figure 4H, middle), no less than the corresponding fUSI signals (Figure 4H, left, p = 0.40, n = 14).

To test whether the bilateral correlations in firing rates fully explain the bilateral correlations in fUSI, we removed the fraction of fUSI signals predicted by filtering the firing rate and examined the residuals. These residuals had a much smaller bilateral covariance than the original fUSI signals, both in visual cortex (paired t test p < 10^−10^, Figure 4D, right) and in hippocampus (p < 10^−4^, Figure 4H, right). They strongly correlated across the fUSI slice (Figure S2), suggesting that they reflect global brain movements and global vascular effects, perhaps related to heartbeats, respiration, and oscillations in the arterial diameter (Drew, 2019; Winder et al., 2017).

## Discussion

Much of brain activity is endogenous—unrelated to external events—so it must be measured in individual trials. Single-trial measurements of brain activity, indeed, are routine with electrophysiology techniques that record local neuronal spikes. However, they are exceedingly difficult with methods that have larger spatial coverage, such as fMRI and EEG. These methods have low signal/noise ratios and thus require recordings to be averaged across trials (event-related analysis) or internal events (e.g., correlation with a seed voxel).

Our results indicate that fUSI in mice can bridge this gap, providing large-scale measurements of brain activity in single trials. By performing simultaneous electrophysiology and fUSI, we were able to establish the relationship between neuronal firing and ultrasound signals on a trial-by-trial, moment-by-moment basis. The results indicate that functional ultrasound signals measured at frequencies below 0.3 Hz strongly correlate with neural activity. This high signal/noise ratio may explain why fUSI signals can even drive brain-machine interfaces (Norman et al., 2021).

We found that fUSI signals bear a simple relationship to neural activity captured by convolution with a standard hemodynamic response function. These results confirm and extend work that related blood signals to fUSI measurements performed separately and averaged across trials (Aydin et al., 2020; Boido et al., 2019). They suggest that the hemodynamic response function measured by fUSI is the same that had been measured optically (Pisauro et al., 2013) and is consistent across mice, stimulus conditions, and brain regions. However, we only tested two regions: visual cortex and hippocampus ; hemodynamic responses might differ elsewhere (Handwerker et al., 2004).

fUSI signals appear noisy because they are variable in time and broadly correlated in space. However, this variability reflects not just measurement error but true structured fluctuations in neuronal firing. Neuronal activity across regions and hemispheres often fluctuates simultaneously (Drew et al., 2019; Fox et al., 2006, 2007; Mohajerani et al., 2010; Shimaoka et al., 2019), and these fluctuations are often associated with changes in brain state and body movement (Drew et al., 2019; Musall et al., 2019; Stringer et al., 2019). Accordingly, our double recordings reveal that fUSI signals match neural activity even when they spread over large portions of the brain, including the opposite hemisphere.

We found a correlation as high as 0.6 between fUSI signals and smoothed firing rates in mice that were mainly awake and alert. The correlation might be even higher during NREM sleep, when the relationship between blood flow and neural activity is thought to be stronger (Turner et al., 2020).

fUSI signals correlated best with the firing of fast-spiking, putative inhibitory neurons. This observation may relate to a specific role of synaptic inhibition in controlling blood flow (Anenberg et al., 2015; Cauli et al., 2004). However, fast-spiking cells are likely to be largely parvalbumin-positive interneurons, whose activation reduces, rather than increase, blood flow (Lee et al., 2021). The high correlation with inhibitory activity seems thus more likely to arise because inhibitory neurons are robust estimators of nearby firing rates (Isaacson and Scanziani, 2011), pooling over more excitatory neurons than those recorded by the probe.

Perhaps, a similar reasoning explains the higher correlations that we observed between fUSI signals and activity in supragranular layers of the cortex. These laminar differences were small but significant and may make it difficult to measure laminar activity with fMRI (Huber et al., 2017).

By releasing our ground-truth spiking data acquired simultaneous with fUSI imaging, we hope to facilitate improvements to the fUSI processing pipeline, which begins with raw Doppler images (sequences of images made of complex numbers measured at ∼500 Hz) and returns estimated blood flow measurements (images of real numbers at ∼3 Hz) through multiple steps (Baranger et al., 2018; Demené et al., 2015; Macé et al., 2011, 2013). Our results confirm that this pipeline is adequate: it yields fUSI signals that closely resemble the underlying firing rates. However, perhaps, it can be further improved. Moreover, one may want to go backward and estimate firing rates from fUSI signals, e.g., as done with widefield calcium fluorescence (Peters et al., 2021). For all this, it is essential to have neuronal spikes as ground-truth data.

We conclude that fUSI signals bear a simple relationship to neuronal firing and accurately reflect this firing in both time and space. We hope that these results will be useful to the increasing numbers of laboratories that use fUSI to reveal brain function.

## STAR★Methods

### Key resources table

**Table:** 

REAGENT or RESOURCE	SOURCE	IDENTIFIER
**Deposited data**		

Mouse Common Coordinate Framework	Allen Institute	https://doi.org/10.1016/j.cell.2020.04.007
Mouse Vascular atlas	Todorov et al., 2020	https://doi.org/10.24433/CO.1402016.v1 http://discotechnologies.org/VesSAP/
Sorted spikes and fUSI videos	This paper	https://figshare.com/projects/Nunez-Elizalde2022/132110

**Experimental models: Organisms/strains**		

C57BL/6J mice	Charles River	https://www.jax.org/strain/000664

**Software and algorithms**		

Matlab	The MathWorks, USA	www.mathworks.com
Functional ultrasound imaging data acquisition package	Alan Urban Technology & Consulting (AUTC)	https://fusi-functional-ultrasound-imaging.com/
Rigbox / Timeline	Bhagat et al., 2020	https://github.com/cortex-lab/Rigbox
FreeSurfer	Fischl, 2012	https://surfer.nmr.mgh.harvard.edu/fswiki/rel7downloads
kilosort2	Pachitariu et al., 2016	https://github.com/MouseLand/Kilosort/releases/tag/v2.0
Phy	Cyrille Rossant	https://github.com/cortex-lab/phy
Coherence analysis	http://nipy.org/nitime/	https://github.com/nipy/nitime
Cross-validated ridge regression	Nunez-Elizalde et al., 2019	https://github.com/gallantlab/tikreg
DeepLabCut	Mathis et al., 2018	https://github.com/DeepLabCut/DeepLabCut
Whisker motion analysis	Stringer et al., 2019	https://github.com/MouseLand/facemap https://github.com/gallantlab/pymoten
Python code to analyze data	This paper	https://doi.org/10.5281/zenodo.6069653

**Other**		

Polymethylpentene film	Goodfellow Cambridge	ME311070
Kwik-Cast	WPI	KWIK-CAST
Video displays	Adafruit	LP097QX1
Fresnel lenses	Wuxi Bohai Optics, China	BHPA220-2-5
Diffusing film	Window Film Company	Frostbite
Neuropixels probes	imec, Belgium	www.neuropixels.org
Ultrasound transducer	Vermon, France	L22-Xtech
Ultrasound system	Verasonics, USA	Vantage 128

### Resource availability

#### Lead contact

Requests for further information should be directed to the lead contact, Matteo Carandini, University College London, m.carandini@ucl.ac.uk.

#### Materials availability

The materials used in this study are available commercially.

### Experimental model and subject details

Experiments were conducted in 5 C57/BL6 mice (4 male, 1 female), 9-12 weeks of age.

### Method details

All experimental procedures were conducted according to the UK Animals Scientific Procedures Act (1986). Experiments were performed at University College London, under a Project License released by the Home Office following appropriate ethics review.

#### Initial surgery

Mice were first implanted with a headplate and cranial window under surgical anesthesia in sterile conditions. Procedures for implanting the headplate are standard in the field (e.g., International Brain Laboratory et al., 2021). The cranial window replaced a dorsal section of the skull (∼8 mm in ML and ∼5 mm in AP) with 90 μm thick ultrasound-permeable polymethylpentene (PMP) film (ME311070, Goodfellow Cambridge Ltd.). The PMP film was then covered with Kwik-Cast (World Precision Instruments, USA), except during imaging sessions. This initial surgery was followed by 5-12 days of recovery, handling, and habituation to the experimental rig.

#### Recording sessions

For each mouse, we determined the location of primary visual cortex (V1) by aligning fUSI images to the Allen Institute’s atlas of the mouse brain (Common Coordinate Framework, Wang et al., 2020) using a vascular atlas as an intermediate reference (Todorov et al., 2020).

In each recording session, we head-fixed the mice by securing the headplate to a post placed 10 cm from three video displays (Adafruit, LP097QX1, 60 Hz refresh rate) arranged at right angles to span 270 deg in azimuth and ∼70 deg in elevation. Fresnel lenses (f = 220 mm, Wuxi Bohai Optics, BHPA220-2-5) were mounted in front of the screens to reduce intensity differences across parts of the screens that are viewed from different angles. The lenses were covered with diffusing film (Frostbite, The Window Film Company) to reduce specular reflections.

We then inserted a Neuropixels probe (Jun et al., 2017) through a hole in the PMP film (0.5 mm radius). The probe described a parasagittal trajectory (posterolateral to anteromedial), at an angle of 28 deg relative to the midline (sagittal plane) and 40 deg relative to the horizontal (axial) plane. In some experiments we introduced a second Neuropixels probe in the opposite hemisphere, along the mirror-symmetric trajectory.

We then covered the PMP film with ultrasound gel and positioned an ultrasound transducer above it (128-element linear array, 100 μm pitch, 8 mm focal length, 15 MHz central frequency, model L22-Xtech, Vermon, France). Doppler signals from the transducer were acquired using an ultrasound system (Vantage 128, Verasonics, USA) controlled by a custom Matlab-based user interface (Alan Urban Consulting) recording continuously at 500 Hz. fUSI acquisition was synchronized with the visual stimulus by recording the TTL pulses of the fUSI frames together with the flickering sync square on the visual stimulus monitor (using TimeLine, Bhagat et al., 2020). A similar method was used to align the Neuropixels recordings, by simultaneous recording external TTL pulses on an additional channel of a Neuropixels probe and on TimeLine.

In each recording session, we moved the ultrasound transducer to cover 3-5 coronal slices. For each slice, we performed two recordings: first, we displayed a gray screen for ∼4 minutes to measure spontaneous activity; second, we presented flashing checkerboards flashing at 2 Hz for ∼8 minutes to measure stimulus-evoked responses. The checkerboards were presented in the left, center, or right screens (one screen at a time). Checkerboard squares had a size of 15 deg and could be white or black. The checkerboard sequence was interspersed with blank trials. The sequence consisted of 40 checkerboards, lasted ∼90 seconds and was repeated 4-5 times.

At the end of the recording session, we slowly extracted the Neuropixels probe from the brain while recording fUSI images from one coronal slice. This movement allowed us to localize the probe’s tip within the fUSI slice, giving us a 2D coronal projection of the probe’s 3D trajectory.

Finally, we acquired a series of coronal fUSI images (a “Y-stack”) from posterior to anterior, spaced 0.1 mm apart. These images were later used to construct a 3D fUSI volume of the brain to facilitate registration with the Allen Atlas and to identify the location of the Neuropixels probe in the fUSI slices.

### Quantification and statistical analysis

#### Processing of ultrasound signals

fUSI signals were computed using standard methods (Macé et al., 2011). The 500 Hz complex-valued Doppler signals were divided into 400 ms chunks that overlapped by 50 ms. Then, each chunk was high-pass filtered with a cut-off of 15 Hz, and its principal components were computed in space and time. The first 15 principal components were then removed, to remove artifacts including those due to brain movement (Demené et al., 2015). A power Doppler image was then computed by squaring the complex-valued signals and averaging them in the central (non-overlapping) 300 ms window, for a final temporal resolution of 3.33 Hz. The voxel time courses were then converted to fractional change relative to the mean of each voxel.

We computed the fUSI signal trace for a region of interest (ROI) by taking the mean of the individual time courses of voxels in the ROI. The individual voxel time courses were normalized to percent signal change units before computing their mean.

fUSI images were manually aligned to a vascular atlas with Allen common coordinate framework (CCF) labels (Todorov et al., 2020). We first registered the 3D volume from each recording session to the vasculature atlas. To this end, we used FreeSurfer (Fischl, 2012) to rotate, shift, and scale the vasculature atlas to match the vasculature features salient in the fUSI 3D volume. Once aligned, the transformation relating the vasculature atlas to the fUSI volume was saved and applied to the vasculature-matched Allen CCF labels. Finally, the Allen CCF labels were resampled to match the spatial resolution of the fUSI volume (100 x 100 x 48 μm^3^), yielding Allen CCF labels for each fUSI voxel.

#### Spatial alignment

To identify brain locations simultaneously traversed by the Neuropixels probe and the fUSI slices, we estimated the 3D trajectory of the Neuropixels probe in the fUSI Y-stack volume. Based on the geometry of the simultaneous recordings, we located the Neuropixels probe insertion site ∼0.2 mm behind the posterior-most fUSI slice. We then reconstructed the Neuropixels probe 3D trajectory so that its 2D coronal projection best matched the 2D coronal projection measured with fUSI *in vivo* during Neuropixels probe extraction*.* This 3D trajectory allowed us to map from Neuropixels probe sites to fUSI voxels in a slice, and vice versa.

While the Neuropixels probe intersects with the fUSI slice plane at one point in space, the fUSI slice has a thickness. This thickness has a full-width at half maximum of ∼300 μm (Brunner et al., 2020) and not larger than 500 μm (Demené et al., 2016). The fUSI voxels and Neuropixels probe sites located 250 μm on either side of the fUSI plane (along the Y-axis) were used for the analyses.

For each recording, we identified the fUSI voxels that were intersected by the Neuropixels probe and used them to define a region of interest (ROI). ROIs for visual cortex and for hippocampus tended to have a similar number of voxels (∼50 voxels). The fUSI signal within the ROI was computed as the mean of the individual voxel time courses.

#### Processing of electrophysiological signals

The electrophysiology data was spike sorted using kilosort2 (Pachitariu et al., 2016) and the resulting output was then manually curated with Phy (github.com/cortex-lab/phy). Manual curation sought to identify clusters corresponding to single- and multi-unit activity and to remove spurious and noisy clusters based on traditional measures such as inter-spike interval, autocorrelation, waveform shape. After spike sorting, single- and multi-unit activity was summed across the electrode sites that traversed the fUSI imaging plane to obtain a single firing rate trace for the ROI. This trace was binned at 300 ms intervals to match the temporal resolution of fUSI signals. To distinguish spikes of putative excitatory and inhibitory neurons we clustered based on spike width (Barthó et al., 2004; Lin et al., 2020).

To analyze the LFP signals we took the LFP output of the Neuropixels probes and separated it into four frequency bands using established methods (Lima et al., 2014).

To identify the Neuropixels probe sites located in visual cortex and in hippocampus, we used the cross-correlation of the multi-unit activity. We divided the Neuropixels probe sites into non-overlapping 100 μm segments and computed their cross-correlation. Sites at the top of the Neuropixels probe corresponded to visual cortex and were strongly correlated with each other. Sites immediately below the visual cortex corresponded to the hippocampus and were strongly correlated with each other.

To obtain ROIs in the fUSI images we identified the fUSI voxels traversed by the Neuropixels probe in visual cortex and hippocampus using the probe’s 3D trajectory and a labeled volume of the standard C57 mouse brain, the Allen Common Coordinate Framework (CCF, Wang et al., 2020). For a ROI in visual cortex or hippocampus we included all voxels that were (1) in the fUSI slice; (2) in the appropriate brain region according to the CCF; and (3) in the Neuropixels probe trajectory.

#### Cross-correlation and coherence

The cross-correlation between firing rate and fUSI signal traces was computed at different delays by shifting the firing rate relative to the fUSI signals (from –5 to +30 s).

Coherence was computed using the multi-taper method (github.com/nipy/nitime). To do this, we used three minutes of firing rate and fUSI signal traces recorded simultaneously during periods of spontaneous activity. We computed the coherence between signals up to 1.667 Hz, the Nyquist limit of our 300 ms sampling interval.

To compute the chance coherence between fUSI signals and firing rate, we randomly and circularly shifted the firing rate and computed its coherence with the original fUSI signal trace. This process was repeated 1,000 times and computing the mean at each frequency. The chance coherence was then computed as the median across recordings for each frequency.

To determine the highest frequency at which firing rate and fUSI signals are coherent, we compared the actual versus chance coherence values across sessions. To do this, we found the frequencies at which actual coherence was above chance. We then identified the highest of these frequencies (0.32 Hz). Above this frequency, the coherence between firing rate and fUSI matches what can be expected by chance.

#### Hemodynamic response function

To estimate the hemodynamic response function relating firing rate to fUSI, we modeled fUSI responses for each recording as a convolution of the firing rate in time with a finite impulse response filter. The optimal filter for each recording was estimated using cross-validated ridge regression (Hoerl and Kennard, 1970) using open-source software (Nunez-Elizalde et al., 2019). To avoid overfitting, the data were split into a training and a test set (75%/25%). Using the training set, the optimal regularization parameter was found independently in each recording using a 5-fold cross-validation procedure twice. The accuracy of the model was assessed by computing the correlation between predicted and actual fUSI signals in the held-out test set. Finally, the hemodynamic response function was estimated for each recording using 100% of the data.

#### Whisker movements and pupil diameter

To assess alertness, we recorded videos of the mouse’s face during our experiments (Figure S2). Using these videos, we quantified pupil size and whisker motion. Pupil diameter was estimated with DeepLabCut (Mathis et al., 2018; Meijer et al., 2020). Whisker motion was estimated following established procedures (Stringer et al., 2019) with publicly available software (github.com/gallantlab/pymoten). The difference between frames was computed for each pixel, yielding a time-by-pixels matrix. The principal components were then computed by concatenating all frames, and the top 10 components were used to compute the total energy over time.

## Data Availability

Processed data (sorted spikes and 3.3 Hz fUSI images) are deposited at Figshare and are publicly available as of the date of publication. The link is listed in the key resources table. Raw data (raw electrical traces and 500 HZ Doppler images) may be obtained from the lead contact upon request. Code to replicate the analyses has been deposited at Zenodo and is publicly available as of the date of publication. The DOI is listed in the key resources table. Any additional information required to reanalyze the data reported in this paper is available from the lead contact upon request.

## Supplemental information

Document S1. Figures S1–S3

Document S2. Article plus supplemental information

## References

[bib1] Anenberg E., Chan A.W., Xie Y., LeDue J.M., Murphy T.H. (2015). Optogenetic stimulation of GABA neurons can decrease local neuronal activity while increasing cortical blood flow. J. Cereb. Blood Flow Metab..

[bib2] Attwell D., Iadecola C. (2002). The neural basis of functional brain imaging signals. Trends Neurosci..

[bib3] Aydin A.K., Haselden W.D., Goulam Houssen Y., Pouzat C., Rungta R.L., Demené C., Tanter M., Drew P.J., Charpak S., Boido D. (2020). Transfer functions linking neural calcium to single voxel functional ultrasound signal. Nat. Commun..

[bib4] Baranger J., Arnal B., Perren F., Baud O., Tanter M., Demené C. (2018). Adaptive spatiotemporal SVD clutter filtering for ultrafast Doppler imaging using similarity of spatial singular vectors. IEEE Trans. Med. Imaging.

[bib5] Barthó P., Hirase H., Monconduit L., Zugaro M., Harris K.D., Buzsáki G. (2004). Characterization of neocortical principal cells and interneurons by network interactions and extracellular features. J. Neurophysiol..

[bib6] Bergel A., Deffieux T., Demené C., Tanter M., Cohen I. (2018). Local hippocampal fast gamma rhythms precede brain-wide hyperemic patterns during spontaneous rodent REM sleep. Nat. Commun..

[bib7] Bergel A., Tiran E., Deffieux T., Demené C., Tanter M., Cohen I. (2020). Adaptive modulation of brain hemodynamics across stereotyped running episodes. Nat. Commun..

[bib8] Bhagat J., Wells M.J., Harris K.D., Carandini M., Burgess C.P. (2020). Rigbox: an open-source toolbox for probing neurons and behavior. eNeuro.

[bib9] Bimbard C., Demene C., Girard C., Radtke-Schuller S., Shamma S., Tanter M., Boubenec Y. (2018). Multi-scale mapping along the auditory hierarchy using high-resolution functional ultrasound in the awake ferret. Elife.

[bib10] Blaize K., Arcizet F., Gesnik M., Ahnine H., Ferrari U., Deffieux T., Pouget P., Chavane F., Fink M., Sahel J.A. (2020). Functional ultrasound imaging of deep visual cortex in awake nonhuman primates. Proc. Natl. Acad. Sci. USA.

[bib11] Boido D., Rungta R.L., Osmanski B.F., Roche M., Tsurugizawa T., Le Bihan D., Ciobanu L., Charpak S. (2019). Mesoscopic and microscopic imaging of sensory responses in the same animal. Nat. Commun..

[bib12] Boynton G.M., Engel S.A., Glover G.H., Heeger D.J. (1996). Linear systems analysis of functional magnetic resonance imaging in human V1. J. Neurosci..

[bib13] Brunner C., Grillet M., Sans-Dublanc A., Farrow K., Lambert T., Macé E., Montaldo G., Urban A. (2020). A platform for brain-wide volumetric functional ultrasound imaging and analysis of circuit dynamics in awake mice. Neuron.

[bib14] Buzsáki G., Anastassiou C.A., Koch C. (2012). The origin of extracellular fields and currents—EEG, ECoG, LFP and spikes. Nat. Rev. Neurosci..

[bib15] Cardoso M.M.B., Lima B., Sirotin Y.B., Das A. (2019). Task-related hemodynamic responses are modulated by reward and task engagement. PLoS Biol..

[bib16] Cauli B., Tong X.K., Rancillac A., Serluca N., Lambolez B., Rossier J., Hamel E. (2004). Cortical GABA interneurons in neurovascular coupling: relays for subcortical vasoactive pathways. J. Neurosci..

[bib17] Deffieux T., Demené C., Pernot M., Tanter M. (2018). Functional ultrasound neuroimaging: a review of the preclinical and clinical state of the art. Curr. Opin. Neurobiol..

[bib18] Demené C., Deffieux T., Pernot M., Osmanski B.F., Biran V., Gennisson J.L., Sieu L.A., Bergel A., Franqui S., Correas J.M. (2015). Spatiotemporal clutter filtering of ultrafast ultrasound data highly increases Doppler and fUltrasound sensitivity. IEEE Trans. Med. Imaging.

[bib19] Demené C., Tiran E., Sieu L.A., Bergel A., Gennisson J.L., Pernot M., Deffieux T., Cohen I., Tanter M. (2016). 4D microvascular imaging based on ultrafast Doppler tomography. Neuroimage.

[bib20] Desjardins A.E., Kiehl K.A., Liddle P.F. (2001). Removal of confounding effects of global signal in functional MRI analyses. Neuroimage.

[bib21] Devor A., Ulbert I., Dunn A.K., Narayanan S.N., Jones S.R., Andermann M.L., Boas D.A., Dale A.M. (2005). Coupling of the cortical hemodynamic response to cortical and thalamic neuronal activity. Proc. Natl. Acad. Sci. USA.

[bib22] Dizeux A., Gesnik M., Ahnine H., Blaize K., Arcizet F., Picaud S., Sahel J.A., Deffieux T., Pouget P., Tanter M. (2019). Functional ultrasound imaging of the brain reveals propagation of task-related brain activity in behaving primates. Nat. Commun..

[bib23] Drew P.J. (2019). Vascular and neural basis of the BOLD signal. Curr. Opin. Neurobiol..

[bib24] Drew P.J., Mateo C., Turner K.L., Yu X., Kleinfeld D. (2020). Ultra-slow oscillations in fMRI and resting-state connectivity: neuronal and vascular contributions and technical confounds. Neuron.

[bib25] Drew P.J., Winder A.T., Zhang Q. (2019). Twitches, blinks, and fidgets: important generators of ongoing neural activity. Neuroscientist.

[bib26] Edelman B.J., Macé E. (2021). Functional ultrasound brain imaging: bridging networks, neurons, and behavior. Biomed. Eng..

[bib27] Ferrier J., Tiran E., Deffieux T., Tanter M., Lenkei Z. (2020). Functional imaging evidence for task-induced deactivation and disconnection of a major default mode network hub in the mouse brain. Proc. Natl. Acad. Sci. USA.

[bib28] Fischl B. (2012). FreeSurfer. Neuroimage.

[bib29] Fox M.D., Snyder A.Z., Vincent J.L., Raichle M.E. (2007). Intrinsic fluctuations within cortical systems account for intertrial variability in human behavior. Neuron.

[bib30] Fox M.D., Snyder A.Z., Zacks J.M., Raichle M.E. (2006). Coherent spontaneous activity accounts for trial-to-trial variability in human evoked brain responses. Nat. Neurosci..

[bib31] Gesnik M., Blaize K., Deffieux T., Gennisson J.L., Sahel J.A., Fink M., Picaud S., Tanter M. (2017). 3D functional ultrasound imaging of the cerebral visual system in rodents. Neuroimage.

[bib32] Haider B., Häusser M., Carandini M. (2013). Inhibition dominates sensory responses in the awake cortex. Nature.

[bib33] Hamel E. (2006). Perivascular nerves and the regulation of cerebrovascular tone. J. Appl. Physiol..

[bib34] Handwerker D.A., Ollinger J.M., D'Esposito M. (2004). Variation of BOLD hemodynamic responses across subjects and brain regions and their effects on statistical analyses. Neuroimage.

[bib35] Heeger D.J., Ress D. (2002). What does fMRI tell us about neuronal activity?. Nat. Rev. Neurosci..

[bib36] Hillman E.M. (2014). Coupling mechanism and significance of the BOLD signal: a status report. Annu. Rev. Neurosci..

[bib37] Hoerl A.E., Kennard R.W. (1970). Ridge regression: biased estimation for nonorthogonal problems. Technometrics.

[bib38] Huber L., Handwerker D.A., Jangraw D.C., Chen G., Hall A., Stüber C., Gonzalez-Castillo J., Ivanov D., Marrett S., Guidi M. (2017). High-resolution CBV-fMRI allows mapping of laminar activity and connectivity of cortical input and output in human M1. Neuron.

[bib39] Iadecola C., Nedergaard M. (2007). Glial regulation of the cerebral microvasculature. Nat. Neurosci..

[bib40] Aguillon-Rodriguez V., Angelaki D., Bayer H., Bonacchi N., Carandini M., Cazettes F., Chapuis G., Churchland A.K., Dan Y., International Brain Laboratory (2021). Standardized and reproducible measurement of decision-making in mice. Elife.

[bib41] Isaacson J.S., Scanziani M. (2011). How inhibition shapes cortical activity. Neuron.

[bib42] Jun J.J., Steinmetz N.A., Siegle J.H., Denman D.J., Bauza M., Barbarits B., Lee A.K., Anastassiou C.A., Andrei A., Aydın Ç. (2017). Fully integrated silicon probes for high-density recording of neural activity. Nature.

[bib43] Katzner S., Nauhaus I., Benucci A., Bonin V., Ringach D.L., Carandini M. (2009). Local origin of field potentials in visual cortex. Neuron.

[bib44] Koekkoek S.K.E., Tbalvandany S.S., Generowicz B.S., van Hoogstraten W.S., de Oude N.L., Boele H.J., Strydis C., Leus G., Bosch J.G., van der Steen A.F.W. (2018).

[bib45] Lee J., Stile C.L., Bice A.R., Rosenthal Z.P., Yan P., Snyder A.Z., Lee J.M., Bauer A.Q. (2021). Opposed hemodynamic responses following increased excitation and parvalbumin-based inhibition. J. Cereb. Blood Flow Metab..

[bib46] Lima B., Cardoso M.M., Sirotin Y.B., Das A. (2014). Stimulus-related neuroimaging in task-engaged subjects is best predicted by concurrent spiking. J. Neurosci..

[bib47] Lin I.-C., Okun M., Carandini M., Harris K.D. (2020). Equations governing dynamics of excitation and inhibition in the mouse corticothalamic network. Preprint at bioRxiv..

[bib48] Logothetis N.K., Pauls J., Augath M., Trinath T., Oeltermann A. (2001). Neurophysiological investigation of the basis of the fMRI signal. Nature.

[bib49] Macé E., Montaldo G., Cohen I., Baulac M., Fink M., Tanter M. (2011). Functional ultrasound imaging of the brain. Nat. Methods.

[bib50] Macé E., Montaldo G., Osmanski B.F., Cohen I., Fink M., Tanter M. (2013). Functional ultrasound imaging of the brain: theory and basic principles. IEEE Trans. Ultrason. Ferroelectr. Freq. Control.

[bib51] Macé É., Montaldo G., Trenholm S., Cowan C., Brignall A., Urban A., Roska B. (2018). Whole-brain functional ultrasound imaging reveals brain modules for visuomotor integration. Neuron.

[bib52] Macey P.M., Macey K.E., Kumar R., Harper R.M. (2004). A method for removal of global effects from fMRI time series. Neuroimage.

[bib53] Martindale J., Mayhew J., Berwick J., Jones M., Martin C., Johnston D., Redgrave P., Zheng Y. (2003). The hemodynamic impulse response to a single neural event. J. Cereb. Blood Flow Metab..

[bib54] Mathis A., Mamidanna P., Cury K.M., Abe T., Murthy V.N., Mathis M.W., Bethge M. (2018). DeepLabCut: markerless pose estimation of user-defined body parts with deep learning. Nat. Neurosci..

[bib55] McGinley M.J., Vinck M., Reimer J., Batista-Brito R., Zagha E., Cadwell C.R., Tolias A.S., Cardin J.A., McCormick D.A. (2015). Waking state: rapid variations modulate neural and behavioral responses. Neuron.

[bib56] Meijer G., Schartner M., Chapuis G., Bonacchi N., Winter O., Svoboda K., International Brain Laboratory (2020). https://shorturl.at/oszR2.

[bib57] Mohajerani M.H., McVea D.A., Fingas M., Murphy T.H. (2010). Mirrored bilateral slow-wave cortical activity within local circuits revealed by fast bihemispheric voltage-sensitive dye imaging in anesthetized and awake mice. J. Neurosci..

[bib58] Murphy K., Birn R.M., Handwerker D.A., Jones T.B., Bandettini P.A. (2009). The impact of global signal regression on resting state correlations: are anti-correlated networks introduced?. Neuroimage.

[bib59] Musall S., Kaufman M.T., Juavinett A.L., Gluf S., Churchland A.K. (2019). Single-trial neural dynamics are dominated by richly varied movements. Nat. Neurosci..

[bib60] Nair D.G. (2005). About being BOLD. Brain Res. Brain Res. Rev..

[bib61] Norman S.L., Maresca D., Christopoulos V.N., Griggs W.S., Demené C., Tanter M., Shapiro M.G., Andersen R.A. (2021). Single-trial decoding of movement intentions using functional ultrasound neuroimaging. Neuron.

[bib62] Nunez-Elizalde A.O., Huth A.G., Gallant J.L. (2019). Voxelwise encoding models with non-spherical multivariate normal priors. Neuroimage.

[bib63] Osmanski B.F., Pezet S., Ricobaraza A., Lenkei Z., Tanter M. (2014). Functional ultrasound imaging of intrinsic connectivity in the living rat brain with high spatiotemporal resolution. Nat. Commun..

[bib64] Pachitariu M., Steinmetz N.A., Kadir S.N., Carandini M., Harris K.D. (2016). Fast and accurate spike sorting of high-channel count probes with KiloSort. Advances in Neural Information Processing Systems.

[bib65] Peters A.J., Fabre J.M.J., Steinmetz N.A., Harris K.D., Carandini M. (2021). Striatal activity topographically reflects cortical activity. Nature.

[bib66] Pisauro M.A., Dhruv N.T., Carandini M., Benucci A. (2013). Fast hemodynamic responses in the visual cortex of the awake mouse. J. Neurosci..

[bib67] Provansal M., Labernède G., Joffrois C., Rizkallah A., Goulet R., Valet M., Deschamps W., Ferrari U., Chaffiol A., Dalkara D. (2021). Functional ultrasound imaging of the spreading activity following optogenetic stimulation of the rat visual cortex. Sci. Rep..

[bib68] Rabut C., Correia M., Finel V., Pezet S., Pernot M., Deffieux T., Tanter M. (2019). 4D functional ultrasound imaging of whole-brain activity in rodents. Nat. Methods.

[bib69] Rabut C., Yoo S., Hurt R.C., Jin Z., Li H., Guo H., Ling B., Shapiro M.G. (2020). Ultrasound technologies for imaging and modulating neural activity. Neuron.

[bib70] Rahal L., Thibaut M., Rivals I., Claron J., Lenkei Z., Sitt J.D., Tanter M., Pezet S. (2020). Ultrafast ultrasound imaging pattern analysis reveals distinctive dynamic brain states and potent sub-network alterations in arthritic animals. Sci. Rep..

[bib71] Reimer J., Froudarakis E., Cadwell C.R., Yatsenko D., Denfield G.H., Tolias A.S. (2014). Pupil fluctuations track fast switching of cortical states during quiet wakefulness. Neuron.

[bib72] Rubin J.M., Adler R.S., Fowlkes J.B., Spratt S., Pallister J.E., Chen J.F., Carson P.L. (1995). Fractional moving blood volume: estimation with power Doppler US. Radiology.

[bib73] Rubin J.M., Bude R.O., Carson P.L., Bree R.L., Adler R.S. (1994). Power Doppler US: a potentially useful alternative to mean frequency-based color Doppler US. Radiology.

[bib74] Sakata S., Harris K.D. (2009). Laminar structure of spontaneous and sensory-evoked population activity in auditory cortex. Neuron.

[bib75] Sans-Dublanc A., Chrzanowska A., Reinhard K., Lemmon D., Nuttin B., Lambert T., Montaldo G., Urban A., Farrow K. (2021). Optogenetic fUSI for brain-wide mapping of neural activity mediating collicular-dependent behaviors. Neuron.

[bib76] Schölvinck M.L., Saleem A.B., Benucci A., Harris K.D., Carandini M. (2015). Cortical state determines global variability and correlations in visual cortex. J. Neurosci..

[bib77] Shaw K., Bell L., Boyd K., Grijseels D.M., Clarke D., Bonnar O., Crombag H.S., Hall C.N. (2021). Neurovascular coupling and oxygenation are decreased in hippocampus compared to neocortex because of microvascular differences. Nat. Commun..

[bib78] Shimaoka D., Steinmetz N.A., Harris K.D., Carandini M. (2019). The impact of bilateral ongoing activity on evoked responses in mouse cortex. eLife.

[bib79] Sieu L.A., Bergel A., Tiran E., Deffieux T., Pernot M., Gennisson J.L., Tanter M., Cohen I. (2015). EEG and functional ultrasound imaging in mobile rats. Nat. Methods.

[bib80] Stringer C., Pachitariu M., Steinmetz N., Reddy C.B., Carandini M., Harris K.D. (2019). Spontaneous behaviors drive multidimensional, brainwide activity. Science.

[bib81] Todorov M.I., Paetzold J.C., Schoppe O., Tetteh G., Shit S., Efremov V., Todorov-Völgyi K., Düring M., Dichgans M., Piraud M. (2020). Machine learning analysis of whole mouse brain vasculature. Nat. Methods.

[bib82] Turner K.L., Gheres K.W., Proctor E.A., Drew P.J. (2020). Neurovascular coupling and bilateral connectivity during NREM and REM sleep. Elife.

[bib83] Urban A., Dussaux C., Martel G., Brunner C., Mace E., Montaldo G. (2015). Real-time imaging of brain activity in freely moving rats using functional ultrasound. Nat. Methods.

[bib84] Wang Q., Ding S.L., Li Y., Royall J., Feng D., Lesnar P., Graddis N., Naeemi M., Facer B., Ho A. (2020). The Allen Mouse Brain Common coordinate framework: a 3D reference atlas. Cell.

[bib85] Winder A.T., Echagarruga C., Zhang Q., Drew P.J. (2017). Weak correlations between hemodynamic signals and ongoing neural activity during the resting state. Nat. Neurosci..

[bib86] Zhang Y.S., Takahashi D.Y., Hady A.E., Liao D.A., Ghazanfar A.A. (2021). Active neural coordination of motor behaviors with internal states. Preprint at bioRxiv..

