# Supplementary material for: Neural correlates of blood flow measured by ultrasound
Source: Neuron. 2022 May 18;110(10):1631–1640.e4. doi: 10.1016/j.neuron.2022.02.012 (PMC9235295; doi:10.1016/j.neuron.2022.02.012)
Supplement: Document S1. Figures S1–S3 [file mmc1.pdf]

Neuron, Volume *110*

Supplemental information

Neural correlates of blood flow measured by ultrasound

Anwar O. Nunez-Elizalde, Michael Krumin, Charu Bai Reddy, Gabriel Montaldo, Alan Urban, Kenneth D. Harris, and Matteo Carandini

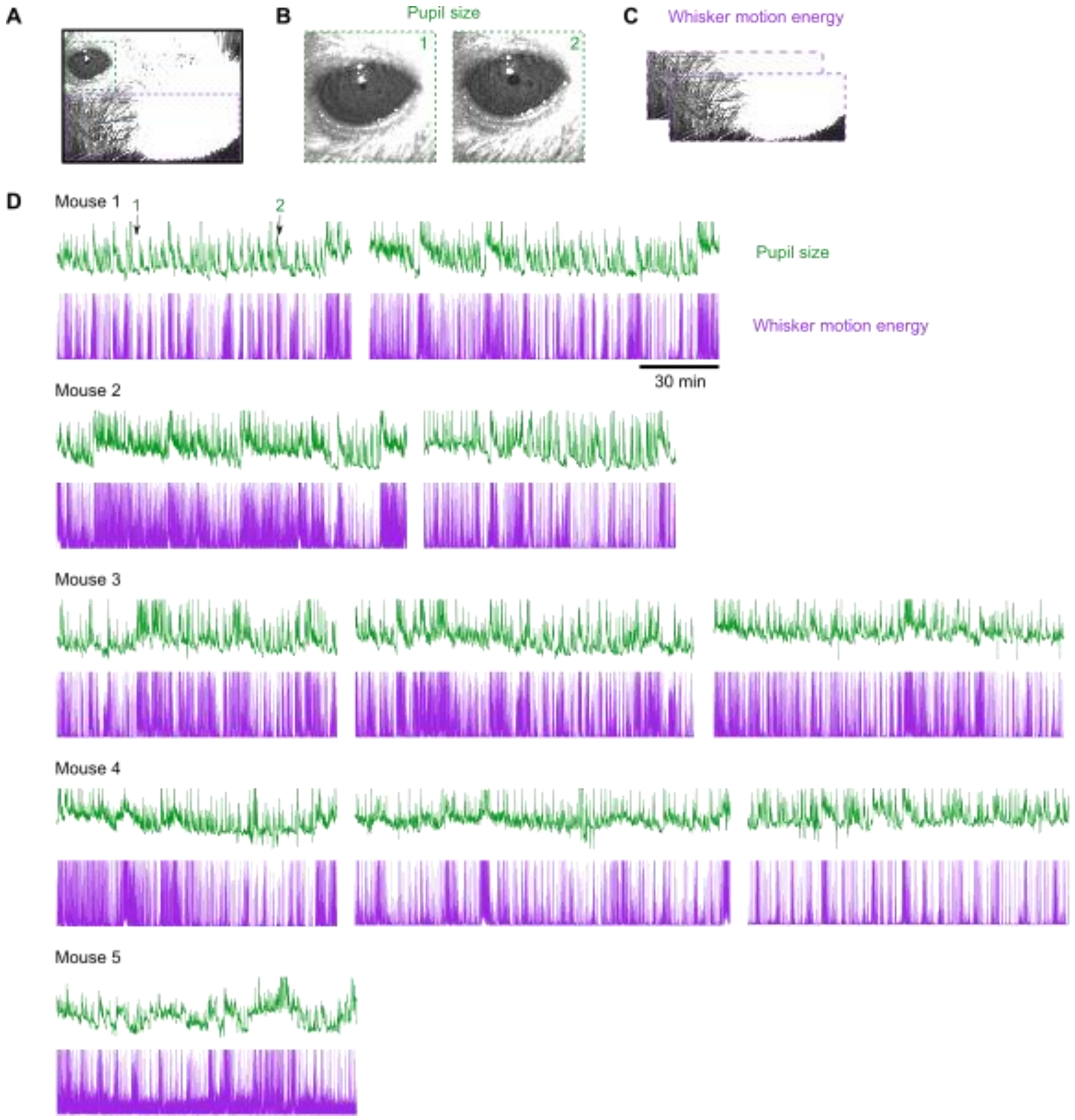

Supplemental Figure 1. **Behavioral monitoring**. Related to Figure 1.
(**A**) Example frame from a camera pointed at the mouse face, showing regions analyzed for eye (*green*) and whiskers (*purple*).
(**B**) Example frames of the eye, used to estimate pupil size, showing a frame with smaller pupil (*1*) and one with larger pupil (*2*).
(**C**) Example frames of the whiskers, used to estimate whisker motion energy.
(**D**) Pupil size (*green*) and whisker motion energy (*purple*) for 11 recording sessions in 5 mice. Arrows 1 and 2 mark the frames in B.

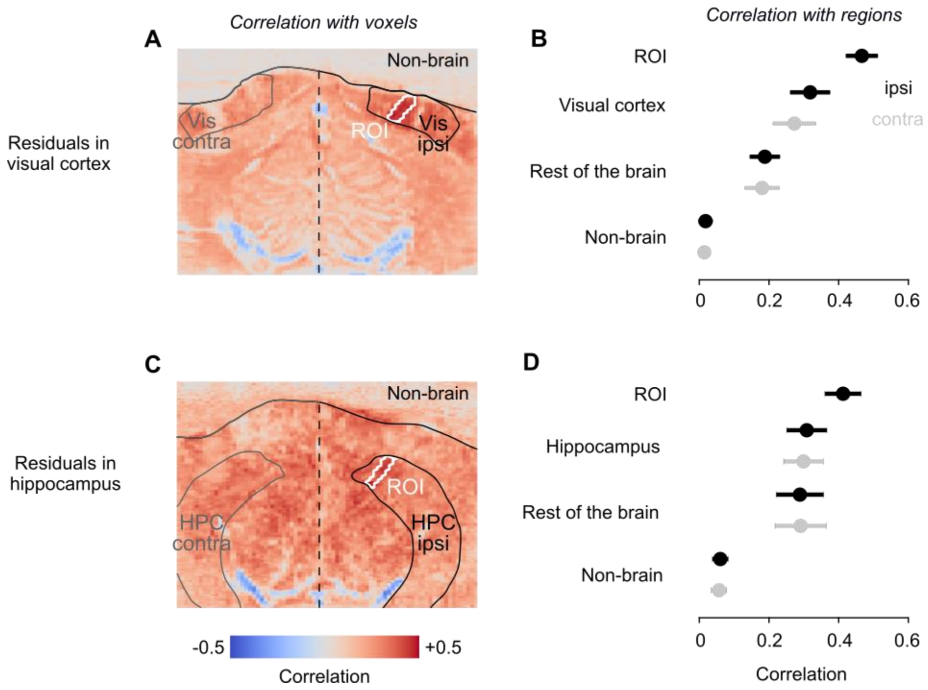

Supplemental Figure 2. **fUSI residuals correlate with the whole brain**. Related to Figure 4.
(**A**) Correlations between fUSI residuals (fUSI signals minus filtered firing rate) in visual cortex with fUSI signals in the whole slice.
(**B**) Correlations with fUSI signals in the ROI, in the rest of visual cortex, in contralateral visual cortex, and in the rest of the brain.
(**C**,**D**) Same, for fUSI residuals in hippocampus.

	Bai Reddy	Carandini	Harris	Krumin	Montaldo	Nunez-Elizalde	Urban
Conceptualization		•	•	•		•	
Methodology	•	•	•	•	•	•	•
Software				•	•	•	•
Formal Analysis				•		•	
Investigation	•			•		•	
Resources		•	•		•		•
Writing – Original Draft		•				•	
Writing – Review & Editing		•	•	•	•	•	•
Visualization		•				•	
Supervision		•	•				
Funding Acquisition		•	•				

Supplemental Figure 3. **Author contributions**. Matrix representation of the information in Author Contributions.
